# Pathways to Prevention for Children of Depressed Mothers: A Review of the Literature and Recommendations for Practice

**DOI:** 10.1155/2012/313689

**Published:** 2012-01-31

**Authors:** Carrie W. Rishel

**Affiliations:** Division of Social Work, West Virginia University, P.O. Box 6830, Morgantown, WV 26506, USA

## Abstract

Maternal depression is one of the most well-documented risk factors for child and adolescent depression, but little work has focused on how to reduce this risk. Although a few interventions have been developed and tested, implementing targeted prevention efforts with depressed mothers and their children is not common practice. The increased risk of depression for children of depressed mothers is so clear, however, professionals can no longer “sit on the sidelines” without initiating specific prevention efforts with this population. To do so requires a paradigm shift—moving from a focus on individual treatment to a prevention approach that engages the entire family as the unit of care. The purpose of this paper is to draw on existing literature to highlight potential “pathways to prevention” for children of depressed mothers. Recommendations for initiating these pathways based on family lifecycle stage, point of contact, and service setting are presented and discussed.

## 1. Introduction

Decades of research has shown that maternal depression is one of the most potent risk factors for child and adolescent depression, (e.g., [1–5]), with lifetime estimates suggesting that half of children growing up with a depressed parent will be diagnosed with a depressive disorder by age 20 [6]. Very little work, however, has focused on how to reduce this risk. The few studies that have tested prevention interventions in children of depressed parents have reported promising results [7, 8], yet a preventative approach with this population is not common practice [9]. A recent report [10] highlights the urgent need to improve prevention of mental, emotional, and behavioral disorders among youth. The report notes that, “interventions before the disorder occurs offer the greatest opportunity to avoid the substantial costs to individuals, families, and society that these disorders entail” (p. 1).

The risk of depression for children of depressed mothers is clear. Children of depressed mothers are up to 6 times more likely than other children to develop depression [11, 12]. More specifically, a recent study reports that 41.5% of children of postnatally depressed mothers experienced depression by age 16 as compared to 12.5% of children of nondepressed mothers [13]. Another recent study demonstrates that maternal depression is one of two key factors to distinguish young children (age 5 and under) with high levels of anxiety and depressive symptoms from other children (the other factor was difficult temperament at 5 months) [14]. With maternal depression arguably one of the most empirically supported risk factors for child and adolescent depression, professionals who work with families and youth can no longer “sit on the sidelines” without initiating targeted prevention efforts with depressed mothers and their children. The purpose of this paper is to draw on existing literature to highlight potential “pathways to prevention” for children of depressed mothers. Recommendations for initiating these pathways based on family lifecycle stage, point of contact (mothers or children), and service setting are presented and discussed.

## 2. Transmission of Risk

As reviewed in Rishel [15], proposed models of risk transmission from depressed mothers to children fall under two overall categories: transmission through a biological or genetic mechanism and transmission through some aspect of mother-child interaction. Although biological mechanisms likely play an important role in risk transmission, they do not fully account for the increased risk of children of depressed mothers [16]. Mother-child interaction has been shown to be a key factor in the development of depression and other mental, emotional, and behavioral problems in children of depressed mothers [17–19]. Suggested mechanisms by which mother-child interaction transmits risk from depressed mother to child include maternal modeling of depressed affect, cognitions, and behavior; reduced positive reinforcement for the child and inconsistent discipline practices; the development of an insecure child attachment; an indirect influence of maternal depression through its detrimental effects on the marital relationship and family functioning [20, 21]. Although these mechanisms involve different aspects of the mother-child relationship, all imply decreased ability of depressed mothers to effectively parent their children. This implication is supported by literature demonstrating increased difficulty in parenting of depressed mothers as compared to nondepressed mothers [12, 17]. Mothers with depression are more likely to display critical and negative behavior toward their children, less likely to display positive or reinforcing behavior, and less likely to display attachment or involvement with their children than are nondepressed mothers [16, 18, 22]. It is also clear that children are negatively affected by poor mother-child interaction, with child affective disorders and behavioral symptoms significantly related to maternal negativity, criticism, and lack of involvement [16, 18].

## 3. A Prevention-Focused Approach

Taking into consideration the clear evidence of the detrimental effect of maternal depression on children, it is time for a paradigm shift—a shift away from the traditional focus on individual treatment and toward a prevention-focused approach to child and adolescent depression. The prevention approach requires moving beyond the medical-based disease model, in which practitioners wait for a child or adolescent to develop clinical depressive symptoms and then provide evidence-based treatment, to a practice philosophy that focuses on the long-term healthy development of youth and seeks to proactively provide resources to support this healthy development [10, 23]. A shift toward prevention-focused practice requires multiple systems involved in the lives of children and families (e.g., primary medical system, specialty mental health system, early childhood services, and education system) to develop effective collaborative relationships that promote proactive implementation of preventive interventions with children of depressed mothers.

Prevention efforts are categorized into three levels: (1) *universal* interventions are provided to the general public or an entire population group; (2) *selective* interventions target those at elevated risk for a particular problem as compared to the general population; (3) *indicated *interventions target high-risk individuals who already demonstrate symptoms of a specific disorder, but do not yet meet clinical criteria [24]. Prevention efforts aimed at reducing the development of depression in children and adolescents of depressed mothers are considered *selective *interventions, as they target youth at elevated risk for depression based on an empirically established risk factor. If, as may sometimes be the case, youth participants of a *selective* intervention already display symptoms of depression (but do not yet meet clinical criteria for a depressive disorder), these prevention efforts may also be considered *indicated *interventions.

Prevention efforts targeting children of depressed mothers are typically psychological and/or educational in nature, focusing on mother-child models of risk transmission, as opposed to biological or genetic ones. While there is considerable evidence for a genetically linked familial component to depression (please see [21, 25] for a review of this literature), prevention efforts targeting genetic risk have not yet been developed. As reviewed in Costello et al. [25], the development of biological prevention efforts has been limited by the lack of studies using twin and cross-fostering designs. It has, therefore, been difficult to separate the influence of genetics and environment on the onset of depression, or examine the impact of gene-environment interactions. The Costello et al. [25] review of the development and natural history of mood disorders provides recommendations for future research to examine genetic vulnerability factors in depression onset. The results of this research will hopefully inform future prevention efforts with children of depressed mothers.

It is important to note here that while this paper focuses on identifying pathways to prevention for children of depressed mothers, much of the discussion may be relevant to children of depressed fathers as well. Although many fewer studies of paternal effects have been conducted, a few preliminary studies in this area suggest that paternal depression negatively impacts children's development [26–29]. A comprehensive discussion of paternal-child risk transmission and potential points of intervention is beyond the scope of this paper. Many of the strategies addressed, however, could be adapted to target paternal-child transmission rather than, or in addition to, maternal-child transmission of risk.

## 4. Review of Prevention Efforts

Despite extensive research documenting the increased risk of psychopathology for children of depressed mothers, surprisingly little prevention research has been conducted with this population [30]. Beardslee and colleagues are the clear leaders in this area and have been developing and testing family-based interventions for children of depressed parents for the past two decades. Their work has compared two family-based interventions, one in which a trained clinician facilitates 6–10 cognitive-based sessions split among child, parent, and family sessions, and the other a 2-session psychoeducational lecture intervention provided to parents in a group format without child participation [31, 32]. These interventions have also been adapted for implementation with low-income and culturally diverse urban families [33]. Outcome research demonstrates positive effects for both interventions, with stronger effects for the clinician-facilitated parent and child intervention [34] that have been sustained for up to three years [35]. More recently, a decrease in internalizing symptoms over a 2.5-year followup has been reported for children in both interventions [7]. While these results are promising, the interventions have not yet been tested in randomized controlled trials [36]. A similar cognitive-based intervention, delivered to adolescents of depressed parents in a group format, has been tested in a randomized trial and shown a significant decrease in incidents of major depressive episodes over a fifteen-month period [7].

 These two interventions, as well as most others delivered to youth considered at increased risk for depression, are based on components of cognitive therapy [37]. Garber [38] notes that other depression prevention strategies have included a focus on coping, social, and communication skills, and parenting, but that a comprehensive prevention program targeting multiple domains of risk and protection has not yet been developed. Both Garber [38] and Merry and Spence [37] conclude that a lack of rigorous efficacy trials of depression prevention hinders wide-scale implementation of depression prevention programs, but agree that positive findings thus far are promising and further research is warranted. Based on their review of the depression prevention literature, Barrera et al. [36] assert that depression prevention is a feasible goal for the 21st century and, moreover, suggest that integrating depression prevention into standard mental health services would greatly reduce the negative impact of depression worldwide.

 Recent systematic reviews of depression prevention efforts have reported mixed results. Authors of a meta-analysis on depression prevention found a weighted mean effect size of 0.22 and estimated that prevention programs could achieve an 11% improvement in depressive symptoms [39]. Another systematic review and meta-analysis of depression prevention programs noted that while effect sizes were small, some studies reported a reduction in the incidence of depressive episodes, which translated to a “number needed to treat” of 10. The authors clarify that this means that 10 youth would need to receive the intervention in order to prevent the onset of depression in one young person over the following year and conclude that these results are very encouraging for depression prevention programs [40]. In an update to this review, authors conclude that results of depression prevention programs are mixed, noting the positive results of several targeted studies, but the failure of attempts to replicate these findings in school and primary care settings, as well as a number of methodological problems in studies to date [37].

Flay et al. [41] describe the process of testing interventions beginning with intervention development, then moving to efficacy testing under controlled conditions, effectiveness trials under real-world conditions, and finally to widespread dissemination. Although it is undoubtedly an important goal for prevention researchers to improve efficacy research in depression prevention prior to conducting effectiveness trials and widespread dissemination [38], there is a danger in thinking practitioners should stick to “practice as usual” in light of the lack of efficacy support to date for depression prevention efforts with children of depressed mothers. The negative impact of depression is so great, and the heighted risk to children of depressed mothers is so clear, that we must initiate prevention efforts in multiple practice domains based on the best available research up to this point. To do so, we must embrace the paradigm shift, which includes moving from the “practice as usual” focus on individual treatment to a prevention approach that engages the entire family as the unit of care. There is no one “right” prevention intervention for all families, but multiple prevention pathways that can be initiated at various stages of the family lifecycle in a variety of service settings. Although cognitive-based interventions have shown great promise, this is just one potential pathway to prevention, one that arguably maintains a focus on the individual. Other pathways need to be identified so that practitioners who interface with families in all types of service settings (e.g., prenatal care, adult primary medical care, specialty mental health services, pediatrics, early childhood services, and educational settings) can integrate depression prevention efforts into their standard work with families, thus a prevention-focused approach becoming the new “practice as usual.”

## 5. Focusing on Mother-Child Interaction

When developing and implementing prevention interventions for children of depressed mothers, the target of change should be the mother-child unit (or family unit), rather than the individual mother or child. One of the leading researchers examining prevention interventions for children of depressed parents makes the case that depression is an interpersonal illness that directly affects parenting and all family relationships; therefore a family-based approach to prevention is necessary [31]. The term, “family-based services” is defined in the field of children's mental health as a broad range of family-focused interventions that support families' engagement with mental health services [42, 43]. Based on the above review of models of risk transmission from mother to child, it is clear that the impact of maternal depression on the mother-child relationship is profound. Facilitating new patterns of mother-child interaction may be one of the key factors in mitigating the risk to children of depressed mothers. Mothers experiencing depression care deeply about its effect on their children. Beardslee [31] reports that in interviews with affectively ill parents, mothers and fathers express worry that they are damaging their children and frustration that these concerns are not systematically addressed in the health care system. Depressed mothers are also willing to seek mental health services for their children, often overcoming numerous logistical barriers to obtaining services, even if they are not willing to accept treatment for themselves [44–46]. When initiating prevention pathways with children of depressed mothers, it is important to capitalize on the genuine concern mothers express for their children by framing interventions in ways that address these concerns.

 Providing effective treatment for depressed mothers as part of any prevention efforts with their children is, of course, ideal. What if, however, mothers are reluctant to accept treatment for themselves but are willing to accept services for their children? Rather than giving up (and perhaps labeling the mother as “resistant” to treatment), could we instead present the intervention as a way of helping her child/children optimize healthy development? Many cognitive-based prevention efforts include psychoeducational components about the impact of parental depression on children as part of family-focused interventions [36]. Prevention efforts must expand upon this base and also address patterns of mother-child interaction (e.g., maternal affect, positive reinforcement) even if treatment directly addressing the maternal depressive disorder or symptoms is not immediately accepted. By first offering these mothers (who may be reluctant to accept individual depression treatment but concerned about problematic behavior in their children) an intervention focused on the mother-child relationship, clinicians may be able to establish the trusting relationship necessary for mothers to accept treatment focusing on their own depression. Of course, when working with mothers struggling with severe forms of depression who may be in danger of harming themselves or their children, all efforts should be made to provide effective pharmacological and/or psychotherapeutic depression treatment immediately.

One promising approach focusing on the mother-child relationship is Parent-Child Interaction Therapy (PCIT), an empirically supported intervention for disruptive behavior disorders in children that have demonstrated significant long-term effects. The original PCIT targets the parent-child relationship through behavioral and play therapy techniques to improve the relationship and teach the parent to establish appropriate limits [47]. This intervention has been recently adapted for treatment of depression in preschoolers, named Parent-Child Interaction Therapy Emotion Development (PCIT-ED), and initial findings indicate a significant decrease in depression severity scores with a large effect size [48]. While not yet tested as a depression prevention intervention for children of depressed mothers, adapting PCIT to this population may be especially beneficial given the impact of maternal depression on the mother-child relationship. An adapted version of PCIT is just one of several potential prevention interventions that could be initiated with depressed mothers and their children. For depressed mothers and their infants, attachment-based interventions such as the ABC program (Attachment and Biobehavioral Catch-up) [49] that aims to help caregivers (for whom providing nurturance does not come naturally) improve their nurturing skills, may be more appropriate. Attachment-based interventions target the mother-child attachment problems that are more common in families of depressed mothers [50]. (For a review of attachment-based interventions for mothers and infants please see [51].) Determining the most appropriate prevention intervention strategy with depressed mothers and their children depends on several key factors, including the family lifecycle stage and service setting.

## 6. Potential Points of Intervention

In determining potential “points of intervention” at which professionals could initiate prevention efforts to halt or reduce the transmission of risk from depressed mother to child, three key family lifecycle stages become apparent: (1) efforts initiated with mother (and/or father or other key family members) during pregnancy, postpartum, or while child is still too young to participate in intervention; (2) efforts initiated with mother and child (and other key family members as appropriate) once child is old enough to participate in intervention; (3) efforts initiated with mother and child (and other key family members as appropriate) after child had started to display signs or symptoms of depression but does not yet meet clinical criteria for diagnosis of a depressive disorder. The specific prevention effort initiated should be based both on the family lifecycle stage at which the practitioner interacts with the family, and on the point of contact, that is whether it is the child or the mother who first presents for services. Mothers are most likely to seek health care services in three specific settings including prenatal care, primary care offices, and in specialty mental health services. Likewise, children are most likely to present for care in three distinct settings including at pediatrician/well-child visits, specialty child mental health services, and early childhood or education settings. As we move forward with efforts to initiate pathways to prevention for children of depressed mothers, we must consider how to best match intervention approaches to the family lifecycle stage, point of contact, and service location at which the practitioner interfaces with the family.

## 7. Pathways to Prevention

**Figure 1:**
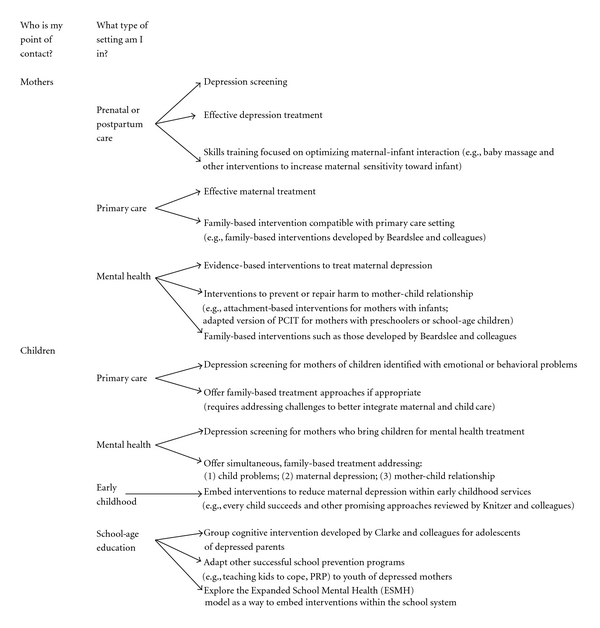
Practitioner-based flow chart to identify pathways to prevention for children of depressed mothers.

Specific pathways to prevention for children of depressed mothers could be initiated at various stages of the family lifecycle and must be appropriate to the point of contact and type of service. Similar to the importance of matching interventions with appropriate stages of change as noted in the Transtheoretical Model of Behavior Change [52], providing interventions that are a good “fit” with the family in terms of lifecycle stage and type of service that has already been sought out and accepted offers to best opportunity for intervention completion and positive impact. Recommendations for initiating prevention pathways when working with depressed mothers and their children are provided below.  Figure 1 provides a practitioner-based flow chart to assist various types of service providers in identifying potential pathways to prevention for children of depressed mothers. Although this is by no means a comprehensive list of potential prevention approaches, it is hoped that this paper will spark further dialogue and research related to targeted prevention efforts for children of depressed mothers.

### 7.1. Mothers as Point of Contact

Mothers experiencing depression or depressive symptoms are most likely to come into contact with practitioners through the obstetrics system, in primary care settings, or when seeking specialty mental health care.

#### 7.1.1. Prenatal and Postpartum Care Settings

Given the high prevalence of depression among women of childbearing age [9], practitioners working with women in prenatal care settings should routinely screen for depression and provide targeted treatment and preventive services. Many mothers, who may not otherwise initiate contact with the health care system, receive regular medical care during the prenatal period. Health care professionals need to capitalize on this period of engagement by utilizing the opportunity to involve women in depression education and treatment [15]. While there are many efforts to ensure screening for postpartum depression becomes common practice [53, 54], it is less apparent that women presenting for prenatal care are adequately screened and treated for depression. Pregnant mothers who screen positive for depressive disorders or symptoms should be provided with effective education and treatment, preferably in the same location where they are receiving prenatal services. There is some evidence that supports providing prevention intervention to pregnant women with elevated depressive symptoms as a way to reduce the incidence of postpartum depression, but results of studies in this area have been mixed [35]. Further research should continue to investigate effective depression prevention in the prenatal period, but education about the impact of maternal depression on children and skill-building opportunities to enhance parenting skills and optimize maternal-infant interaction should also be provided for pregnant mothers with elevated depressive symptoms. Baby massage is one nontraditional intervention that has been shown to be highly effective in improving depressed mothers' sensitivity toward their infants [55]. Proactively teaching depressed mothers ways to appropriately respond to and interact with their infants, as well as stressing the importance of this interaction for healthy child development, is one way that prenatal and postpartum caregivers may be able to initiate a pathway to prevention for children of depressed mothers. These families will, most likely, require further intervention as they move through the family lifecycle, but empowering depressed mothers to learn and become confident in appropriate interaction skills with their infants is an important first step.

#### 7.1.2. Primary Care Settings

Another place many depressed mothers will interact with the health care system is in primary medical settings. While many primary care professionals receive some training in depression and other mood disorders, most do not feel adequately prepared to treat adult depression [9]. Developing better training in maternal depression, effective treatment and its impact on children for primary health professionals, the ones many depressed mothers first turn to for help, is a key factor in moving toward a prevention approach for children of depressed mothers. The family-based interventions developed by Beardslee and colleagues were designed to be compatible with primary care settings [31], and their documented positive outcomes suggest these may be good interventions of choice for primary health professionals.

#### 7.1.3. Specialty Mental Health Settings

Depressed mothers who have sought and accepted treatment with mental health practitioners should be offered evidence-based interventions to effectively treat their depressive symptoms or disorders. Although much research has focused on the link between maternal depression and child mental health problems, very few studies have specifically examined whether effective treatment for depressed mothers lessens the risk for their children [15]. Results of one recent study in this area indicate that remission of maternal depression after three months of medication treatment was significantly associated with a decrease in children's diagnoses and symptoms, while persistence of maternal depression was associated with an increase in children diagnoses and symptoms [56]. Another recent study demonstrates that interpersonal psychotherapy for depressed mothers decreased depressive symptoms in both mothers and their children [57]. Most recently, Wickramaratne and colleagues demonstrated decreased psychiatric symptoms in children whose mothers were successfully treated for depression in the STAR*D study (Sequenced Treatment Alternatives to Relieve Depression) [58]. These studies suggest that effectively treating maternal depression may be one way to halt or reduce the transmission of risk from depressed mother to child, and therefore, a critically important pathway to prevention for children of depressed mothers.

Individual treatment of the mother, however, may not always be enough to change habitual mother-child interaction patterns that may have developed over months or years. Adult mental health services are often totally separate from child mental health services [59]. This fragmented system results in missed opportunities to effectively address mother-child interaction problems that may stem from maternal depression. Most interventions offered to depressed mothers target only the adult depression; they do not address the impact depression has on the parental role or include strategies to prevent or repair harm to the mother-child relationship [60]. Adult and child mental health providers should seek ways to collaborate in the treatment of depressed mothers and their children. Knitzer et al. [60, page 6] call this a “two-fer” where “treatment for the [mother] becomes prevention or early intervention for the child (and for the parent-child relationship).” An adapted version of Parent-Child Interaction Therapy, as discussed above, may be one intervention strategy that could be developed and implemented as a collaborative model in specialty mental health settings as a way to facilitate healthy changes in mother-child interaction patterns with depressed mothers and their children. The family-based interventions developed by Beardslee and colleagues could also be offered to depressed mothers seeking treatment from specialty mental health providers.

### 7.2. Children as Point of Contact

Many mothers experiencing depression do not seek treatment for themselves. For this reason, children of depressed mothers may be the most likely point of contact for families struggling with maternal depression. Children of depressed mothers may come into contact with health and social service providers through primary care settings, specialty mental health settings, early childhood services, or education settings.

#### 7.2.1. Primary Care Settings

Parents who do not seek medical care for themselves may be most likely to be involved with the health care system during the prenatal and pediatric care period [15]. Therefore, pediatricians and family practitioners could play a key role in offering family-based services to depressed mothers and their children [9]. During regularly scheduled well-child visits, pediatricians and family practitioners may identify child emotional or behavioral problems, or difficulties in the mother-child relationship. At this point, the mothers of these children should be screened for depression and offered family treatment approaches as appropriate. Weissman and Olfson [9] note that while most pediatricians believe recognizing maternal depression is part of their responsibilities, very few have been trained in adult mental health interviewing techniques or are familiar with depression screening instruments. This represents a challenge that must be addressed in order to better integrate maternal and child care. Some suggestions provided by Weissman and Olfson [9] include offering brief depression screens to parents whose children display psychiatric symptoms, expanding staff to include primary care clinicians trained in the treatment of mental health problems, improving referral mechanisms to improve access from pediatric offices to adult mental health services, and expanding the collaborative care model for depressed parents and their children. Similar suggestions are supported in recent clinical guidelines commissioned by the National Institute for Health and Clinical Excellence in the United Kingdom [61] that highlight the importance of exploring the possibility of parental depression when a young person is diagnosed with depression. Further research and pilot programs should explore these options as ways to support family-based prevention and treatment approaches for families experiencing maternal depression that are identified through pediatric providers.

#### 7.2.2. Specialty Mental Health Settings

When children are brought by parents for specialty mental health services, it is quite likely that the mother is also struggling with depression or other psychiatric problem [46]. Interviews with mothers bringing children for mental health care indicate that these mothers believe their own depressive symptoms will improve if their children's symptoms improve [44]. Preliminary evidence supports this belief, demonstrating that mothers whose children improved in treatment were more likely to demonstrate a reduction in depressive symptoms than mothers whose children did not improve [62]. Offering simultaneous treatment, however, in which the child problems, maternal depression, and the mother-child relationship are all addressed, should become the new standard of care. Since many depressed mothers bringing their children for services are reluctant to follow up on referrals and accept individual treatment for themselves [44], it is imperative that family-based approaches (e.g., the Beardslee model or adapted version of Parent-Child Interaction Therapy) be offered at the same location, and preferably by the same providers, that the mother originally sought services for her child. Presenting these approaches to the mother as a way to effectively help her child or children is also likely a key factor to promote their acceptance and success.

#### 7.2.3. Early Childhood Services

Early childhood services represent a promising, but underexplored, setting for initiating pathways to prevention for children of depressed mothers [24]. Knitzer et al. [60] suggest that embedding interventions designed to reduce maternal depression and its impact on young children into early childhood programs, such as home-visiting and Early Head Start programs, could be a powerful strategy to enhance outcomes for children and families. They also note the importance of providing family-based interventions that address the mother-child unit rather than the traditional mental health approach of individual treatment. Other work examining prevention of child mental health problems supports further investment into the development and testing of interventions in early childhood programs and services. Based on a meta-analysis of prevention programs, Durlak and Wells [63, 64] conclude that offering intensive family-oriented services before children enter kindergarten or first grade is a potentially promising strategy for preventing mental health problems in youth. While health care settings may offer some advantages in familiarity with intervention delivery, early childhood programs have the potential to reach families who may be reluctant to seek and accept services through the traditional health care system. In their report addressing maternal depression and its impact on young children, Knitzer et al. [60] highlight emerging efforts across the United States to provide innovative prevention services to depressed mothers and their children. One of these programs, *Every Child Succeeds*, is an approach that integrates cognitive behavioral therapy into three different home visiting models and has shown promising initial results, including reduced maternal depression and improved child functioning. Other promising programs are also reviewed in the Knitzer et al. [60] report. Future work should continue to investigate the feasibility and effectiveness of embedding prevention interventions for children of depressed mothers into early childhood services.

#### 7.2.4. Education Settings

Older children and adolescents of depressed mothers, who might already be displaying symptoms of their own depression, may first come to the attention of professionals in the school setting. These children may be referred to the school guidance counselor, social worker, or psychologist by teachers who notice a change in behavior or difficulty functioning in school. Or, older children and adolescents may seek out these school-based professionals on their own. Clarke et al. [8] have reported positive effects of a group cognitive intervention for preventing depression in adolescents of depressed parents. The same prevention intervention has been delivered in a school setting to at-risk adolescents who displayed elevated depressive symptoms and shown positive effects [65]. Replicating this intervention with older children and adolescents of depressed parents who are identified in a school setting seems to be a logical first step in initiating a pathway to prevention for this population. While other school-based depression prevention programs for adolescents have been shown to be effective, such as the Teaching Kids to Cope Program (TKC) [66] and the Penn Resiliency Program (PRP) [67], these interventions did not directly target adolescents of depressed mothers. It is possible, however, that these and other effective school-based adolescent prevention programs could be adapted to address the unique needs of this population. This is an issue that could be explored in future research.

Building upon the national movement throughout the United States to expand school-based mental health programs would be one way to enhance capacity to provide prevention intervention in the school system. These programs, called expanded school mental health (ESMH), provide comprehensive mental health care to youth in the school setting. Prevention efforts through ESMH programs, however, remain underdeveloped [68]. Prevention researchers, mental health providers, and school personnel should continue to seek ways to collaborate and integrate prevention interventions for children of depressed mothers into school-based mental health programs. Similar to embedding interventions for young children into early childhood services, integrating prevention interventions for older children and adolescents into the school system reduces the stigma of seeking services and offers the opportunity to reach many more youth than may be reached through the health care system alone.

### 7.3. Key Factors for Initiating Pathways to Prevention

Regardless of the specific service setting in which a practitioner interacts with depressed mothers and their children, several key factors should be considered when attempting to initiate pathways to prevention. First, referral for services, while common practice, is not a highly effective strategy. Previous work shows that depressed mothers are often reluctant to accept services for themselves, and therefore, do not follow through with referrals to external services [44, 61]. Providers in all of the above-discussed service settings where depressed mothers are likely to seek services for themselves or their children should be trained to implement family-based approaches to initiate pathways to prevention for children of depressed mothers. Successfully, implementing this more holistic approach requires embracing a paradigm shift toward prevention and family-centered care, one that will necessitate increased collaboration among various types of providers and funders.

 A second key factor to consider when initiating pathways to prevention for children of depressed mothers is that the focus, for mothers, may need to initially be on their children. Mothers overcome many barriers to seek services for their children when they perceive their children need help [44] and may be willing to accept family-based intervention approaches if providers focus their explanation of these approaches on how they will benefit their children. A final factor to consider, especially for policy makers and program planners, is that integration of prevention interventions into primary care settings is crucial. There is much less stigma involved for families when seeking services through primary care settings, as compared to specialty mental health settings, and therefore, these services are more likely to be utilized. To successfully move to a prevention approach for children of depressed mothers, prevention researchers should expand efforts to develop and test prevention interventions offered and delivered in prenatal/postpartum, pediatric, and adult primary care settings, and mental health and primary care providers should continue efforts to collaborate and colocate services, perhaps building upon the collaborative care model that includes mechanisms to facilitate communication and information sharing between primary care and mental health clinicians [9].

## 8. Recommendations for an Integrated Approach

Most prevention interventions targeted at children of depressed parents are considered *selective* interventions; these interventions are aimed at a particular group who are at elevated risk for the development of depression based on a certain risk factor. Fully embracing the move to a prevention paradigm, however, requires an integrated approach to prevention—one that combines universal, selective, and indicated prevention approaches into a comprehensive prevention plan. The Institute of Medicine's (IOM) Mental Health Intervention Spectrum provides a framework to distinguish universal, selected, and indicated prevention from treatment [69]. Applying this framework to the development of a comprehensive prevention plan for children of depressed mothers would assist researchers, practitioners, policy makers, and program planners to implement strategies that could help break the intergenerational cycle of depression. Universal interventions, which are provided to an entire population group, could include education about the impact of maternal depression and the mother-child relationship on children in all prenatal and pediatric care settings. Education and awareness activities could also be provided through early childhood services. Selective interventions, which could include the interventions developed by Beardslee and colleagues, attachment-based interventions, and an adapted version of Parent-Child Interaction Therapy, as well as others, would be offered to families once maternal depression was identified* in the location at which they are already receiving services*. Finally, indicated interventions would be offered to families if children of depressed mothers were already displaying symptoms of depression themselves. These could include the “Coping with Stress Course” developed by Clarke and colleagues [8], a group cognitive intervention for prevention of depression in adolescents and PCIT-ED (Parent-Child Interaction Therapy Emotion Development), which has shown preliminary evidence as a promising treatment for preschoolers with depression [48]. All indicated approaches should focus on simultaneous treatment using a family-based model of care, rather than reverting to the traditional model of treatment focused on the individual and should specifically address parenting and the mother-child relationship as well as the depressive disorders and/or symptoms of mother and child.

## 9. Conclusion

The World Health Organization (WHO) reports that major depression is the number one cause of disability worldwide [70]. Depression is especially prevalent in women of childbearing years [9], resulting in millions of children throughout the world who have mothers struggling with depression. The risks to children of maternal depression are well documented, yet little work has focused on how to reduce this risk. Advocates of prevention note that the traditional individual-focused treatment approach is an insufficient strategy when there is a high prevalence of a disorder [71]. Given the body of evidence that documents the marked increased risk of depression for children of depressed mothers, it is indefensible not to develop a comprehensive prevention approach for this population. Shifting the standard of care from individual-focused treatment to family-focused prevention requires collaboration among researchers, practitioners, and policy makers, as well as collaboration across professions, in order to provide targeted interventions in the multiple settings in which depressed mothers and their children seek out services. By proactively developing an integrated and comprehensive strategy, professionals who work with families in various settings will have the ability to initiate pathways to prevention for children of depressed mothers. The millions of children affected by maternal depression deserve nothing less.

## References

[1] Beardslee WR, Keller MB, Seifer R (1996). Prediction of adolescent affective disorder: effects of prior parental affective disorders and child psychopathology. *Journal of the American Academy of Child and Adolescent Psychiatry*.

[2] Lieb R, Isensee B, Höfler M, Pfister H, Wittchen HU (2002). Parental major depression and the risk of depression and other mental disorders in offspring: a prospective-longitudinal community study. *Archives of General Psychiatry*.

[3] Spence SH, Najman JM, Bor W, O’Callaghan MJ, Williams GM (2002). Maternal anxiety and depression, poverty and marital relationships factors during early childhood as predictors of anxiety and depressive symptoms in adolescence. *Journal of Child Psychology and Psychiatry and Allied Disciplines*.

[4] Turney K (2011). Chronic and proximate depression among mothers: implications for child well-being. *Journal of Marriage and Family*.

[5] Weissman MM, Warner V, Wickramaratne P, Moreau D, Olfson M (1997). Offspring of depressed parents: 10 years later. *Archives of General Psychiatry*.

[6] Beardslee WR, Podorefsky D (1988). Resilient adolscents whose parents have serious affective and other psychiatric disorders: the importance of self-understanding and relationships. *The American Journal of Psychiatry*.

[7] Beardslee WR, Gladstone TR, Wright EJ, Cooper AB (2003). A family-based approach to the prevention of depressive symptoms in children at risk: evidence of parental and child change. *Pediatrics*.

[8] Clarke GN, Hornbrook M, Lynch F (2001). A randomized trial of a group cognitive intervention for preventing depression in adolescent offspring of depressed parents. *Archives of General Psychiatry*.

[9] Weissman MM, Olfson M (2009). Translating intergenerational research on depression into clinical practice. *Journal of the American Medical Association*.

[10] O’Connell ME, Boat T, Warner KE (2009). *Preventing Mental, Emotional, and Behavioral dDisorders among Young People: Progress and Possibilities*.

[11] Beardslee WR, Versage EM, Gladstone TRG (1998). Children of affectively Ill parents: a review of the past 10 years. *Journal of the American Academy of Child and Adolescent Psychiatry*.

[12] Downey G, Coyne JC (1990). Children of depressed parents: an integrative review. *Psychological Bulletin*.

[13] Murray L, Arteche A, Fearon P, Halligan S, Goodyer I, Cooper P (2011). Maternal postnatal depression and the development of depression in offspring Up to 16 years of age. *Journal of the American Academy of Child and Adolescent Psychiatry*.

[14] Coté SM, Boivin M, Liu X, Nagin DS, Zoccolillo M, Tremblay RE (2009). Depression and anxiety symptoms: onset, developmental course and risk factors during early childhood. *Journal of Child Psychology and Psychiatry and Allied Disciplines*.

[15] Rishel CW, Quinn JK, Zambini IG (2008). The relationship between maternal and child mental health: implications for practice and policy. *Family Relations: 21st Century Issues and Challenges*.

[16] Jacob T, Johnson SL (1997). Parent-child interaction among depressed fathers and mothers: impact on child functioning. *Journal of Family Psychology*.

[17] Burbach DJ, Borduin CM (1986). Parent-child relations and the etiology of depression: a review of methods and findings. *Clinical Psychology Review*.

[18] Burge D, Hammen C (1991). Maternal communication: predictors of outcome at follow-up in a sample of children at high and low risk for depression. *Journal of Abnormal Psychology*.

[19] Cummings EM, Davies PT (1994). Maternal depression and child development. *Journal of Child Psychology and Psychiatry and Allied Disciplines*.

[20] Gelfand DM, Teti DM (1990). The effects of maternal depression on children. *Clinical Psychology Review*.

[21] Goodman SH, Gotlib IH (1999). Risk for psychopathology in the children of depressed mothers: a developmental model for understanding mechanisms of transmission. *Psychological Review*.

[22] Gordon D, Burge D, Hammen C, Adrian C, Jaenicke C, Hiroto D (1989). Observations of interactions of depressed women with their children. *The American Journal of Psychiatry*.

[23] McCave E, Rishel CW (2011). Prevention as an explicit part of the social work profession: a systematic investigation. *Advances in Social Work*.

[24] Rishel CW (2007). Evidence-based prevention practice in mental health: what is it and how do we get there?. *The American Journal of Orthopsychiatry*.

[25] Costello EJ, Pine DS, Hammen C (2002). Development and natural history of mood disorders. *Biological Psychiatry*.

[26] Brennan PA, Hammen C, Katz AR, Le Brocque RM (2002). Maternal depression, paternal psychopathology, and adolescent diagnostic outcomes. *Journal of Consulting and Clinical Psychology*.

[27] Cummings EM, Keller PS, Davies PT (2005). Towards a family process model of maternal and paternal depressive symptoms: exploring multiple relations with child and family functioning. *Journal of Child Psychology and Psychiatry and Allied Disciplines*.

[28] Kane P, Garber J (2004). The relations among depression in fathers, children’s psychopathology, and father-child conflict: a meta-analysis. *Clinical Psychology Review*.

[29] Ramchandani P, Stein A, Evans J, O’Connor TG (2005). Paternal depression in the postnatal period and child development: a prospective population study. *The Lancet*.

[30] Boyd RC, Diamond GS, Bourjolly JN (2006). Developing a family-based depression prevention program in urban community mental health clinics: a qualitative investigation. *Family Process*.

[31] Beardslee WR (1998). Prevention and the clinical encounter. *The American Journal of Orthopsychiatry*.

[32] Beardslee WR, Gladstone TRG (2001). Prevention of childhood depression: recent findings and future prospects. *Biological Psychiatry*.

[33] Podorefsky DL, McDonald-Dowdell M, Beardslee WR (2001). Adaptation of preventive interventions for a low-income, culturally diverse community. *Journal of the American Academy of Child and Adolescent Psychiatry*.

[34] Beardslee WR, Salt P, Porterfield K (1993). Comparison of preventive interventions for families with parental affective disorder. *Journal of the American Academy of Child and Adolescent Psychiatry*.

[35] Beardslee WR, Wright E, Rothberg PC, Salt P, Versage E (1996). Response of families to two preventive intervention strategies: long-term differences in behavior and attitude change. *Journal of the American Academy of Child and Adolescent Psychiatry*.

[36] Barrera AZ, Torres LD, Mũoz RF (2007). Prevention of depression: the state of the science at the beginning of the 21st Century. *International Review of Psychiatry*.

[37] Merry SN, Spence SH (2007). Attempting to prevent depression in youth: a systematic review of the evidence. *Early Intervention in Psychiatry*.

[38] Garber J (2008). Prevention of depression: are we there yet?. *Clinical Psychology: Science and Practice*.

[39] Jané-Llopis E, Hosman C, Jenkins R, Anderson P (2003). Predictors of efficacy in depression prevention programmes: meta-analysis. *The British Journal of Psychiatry*.

[40] Merry S, McDowell H, Hetrick S, Bir J, Muller N (2004). Psychological and/or educational interventions for the prevention of depression in children and adolescents. *Cochrane Database of Systematic Reviews*.

[41] Flay BR, Biglan A, Boruch RF (2005). Standards of evidence: criteria for efficacy, effectiveness and dissemination. *Prevention Science*.

[42] Hoagwood KE (2005). Family-based services in children’s mental health: a research review and synthesis. *Journal of Child Psychology and Psychiatry and Allied Disciplines*.

[43] Olin SS, Hoagwood KE, Rodriguez J (2010). The application of behavior change theory to family-based services: improving parent empowerment in children’s mental health. *Journal of Child and Family Studies*.

[44] Anderson CM, Robins CS, Greeno CG, Cahalane H, Copeland VC, Andrews RM (2006). Why low income mothers do not engage with the formal mental health system: a qualitative study of perceptual barriers to care. *Qualitative Health Research*.

[45] Rishel CW, Greeno CG, Marcus SC, Anderson C (2006). Effect of maternal mental health problems on child treatment response in community-based services. *Psychiatric Services*.

[46] Rishel CW, Greeno CG, Marcus SC (2006). Impact of maternal mental health status on child mental health treatment outcome. *Community Mental Health Journal*.

[47] Hood KK, Eyberg SM (2003). Outcomes of parent-child interaction therapy: mothers’ reports of maintenance three to six years after treatment. *Journal of Clinical Child and Adolescent Psychology*.

[48] Lenze SN, Pautsch J, Luby J (2011). Parent-child interaction therapy emotion development: a novel treatment for depression in preschool children. *Depression and Anxiety*.

[49] Dozier M, Lindhiem O, Ackerman JP, Berlin LJ, Ziv Y, Amaya-Jackson L, Greenberg MT (2005). Attachment and behavioral catch-up: an intervention targeting empirically identified needs of foster infants. *Enhancing Early Attachments: Theory, Research, Intervention, and Policy*.

[50] Wan MW, Green J (2009). The impact of maternal psychopathology on child-mother attachment. *Archives of Women’s Mental Health*.

[51] Barlow J, McMillan AS, Kirkpatrick S, Ghate D, Barnes J, Smith M (2010). Health-led unterventions in the early years to enhance infant and maternal mental health: a review of reviews. *Child and Adolescent Mental Health*.

[52] Prochaska JO, DiClemente CC, Miller WR, Heather N (1986). Toward a comprehensive model of change. *Treating Addictive Behaviors: Processes of Change*.

[53] Earls MF, Siegel BS, Dobbins MI (2010). Incorporating recognition and management of perinatal and postpartum depression into pediatric practice. *Pediatrics*.

[54] Georgiopoulos AM, Bryan TL, Wollan P, Yawn BP (2001). Routine screening for postpartum depression. *Journal of Family Practice*.

[55] Kersten-Alvarez LE, Hosman CMH, Riksen-Walraven JM, Van Doesum KTM, Hoefnagels C (2011). Which preventive interventions effectively enhance depressed mothers’ sensitivity? A meta-analysis. *Infant Mental Health Journal*.

[56] Weissman MM, Pilowsky DJ, Wickramaratne PJ (2006). Remissions in maternal depression and child psychopathology. *Journal of the American Medical Association*.

[57] Swartz HA, Frank E, Zuckoff A (2008). Brief interpersonal psychotherapy for depressed mothers whose children are receiving psychiatric treatment. *The American Journal of Psychiatry*.

[58] Wickramaratne P, Gameroff MJ, Pilowsky DJ (2011). Children of depressed mothers 1 year after remission of maternal depression: findings from the STAR∗D-child study. *The American Journal of Psychiatry*.

[59] Fellin P (1996). *Mental Health and Mental Illness: Policies, Programs, and Services*.

[60] Knitzer J, Theberge S, Johnson K (2008). *Reducing Maternal Depression and Its Impact on Young Children: Toward a Responsive Early Childhood Policy Framework*.

[61] Depression in children and young people: identification and management in primary, community and secondary care. *National clinical practice guideline*.

[62] Rishel CW, Greeno CG, Anderson C (2008). The relationship between maternal and child symptom change in community mental health. *Community Mental Health Journal*.

[63] Durlak JA, Wells AM (1997). Primary prevention mental health programs for children and adolescents: a meta-analytic review. *The American Journal of Community Psychology*.

[64] Durlak JA, Wells AM (1997). Primary prevention mental health programs: the future is exciting. *The American Journal of Community Psychology*.

[65] Clarke GN, Hawkins W, Murphy M, Sheeber LB, Lewinsohn PM, Seeley JR (1995). Targeted prevention of unipolar depressive disorder in an at-risk sample of high school adolescents: A randomized trial of a group cognitive intervention. *Journal of the American Academy of Child and Adolescent Psychiatry*.

[66] Puskar K, Sereika S, Tusaie-Mumford K (2003). Effect of the Teaching Kids to Cope (TKC) program on outcomes of depression and coping among rural adolescents. *Journal of Child and Adolescent Psychiatric Nursing*.

[67] Gillham JE, Hamilton J, Freres DR, Patton K, Gallop R (2006). Preventing depression among early adolescents in the primary care setting: a randomized controlled study of the Penn Resiliency Program. *Journal of Abnormal Child Psychology*.

[68] Tashman NA, Weist MD, Acosta O, Bickman NL, Grady M, Nabors L (2000). Toward the integration of prevention research and expanded school mental health programs. *Children’s Services*.

[69] Weisz JR, Sandler IN, Durlak JA, Anton BS (2005). Promoting and protecting youth mental health through evidence-based prevention and treatment. *The American Psychologist*.

[70] Murray CJL, Lopez AD (1996). *The Global Burden of Disease: Summary*.

[71] Albee GW (2005). Call to revolution in the prevention of emotional disorders. *Ethical Human Psychology and Psychiatry*.

